# A fast algorithm for estimating transmission probabilities in QTL detection designs with dense maps

**DOI:** 10.1186/1297-9686-41-50

**Published:** 2009-11-17

**Authors:** Jean-Michel Elsen, Olivier Filangi, Hélène Gilbert, Pascale Le Roy, Carole Moreno

**Affiliations:** 1 INRA, SAGA, BP27, 31326 Castanet Tolosan cedex, France; 2 INRA, GARen, Agrocampus, 35000 Rennes, France; 3 INRA, GABI, 78352 Jouy en Josas cedex, France

## Abstract

**Background:**

In the case of an autosomal locus, four transmission events from the parents to progeny are possible, specified by the grand parental origin of the alleles inherited by this individual. Computing the probabilities of these transmission events is essential to perform QTL detection methods.

**Results:**

A fast algorithm for the estimation of these probabilities conditional to parental phases has been developed. It is adapted to classical QTL detection designs applied to outbred populations, in particular to designs composed of half and/or full sib families. It assumes the absence of interference.

**Conclusion:**

The theory is fully developed and an example is given.

## Background

Experimental designs used for mapping QTL in livestock based on linkage analysis techniques generally comprise two or three generations. The younger generation consists of large offsprings (either half sib only or mixture of half and full sib) measured on quantitative traits to be dissected. This generation and in most cases their parents are genotyped for a set of molecular markers. Genotyping an older generation (the grand parents) helps the determination of parents' phases, an information essential to linkage analysis. QTL detection is a multiple step procedure. First the parental phases must be determined from grand parental and/or progeny genotype information, either looking for their most probable phase, or building all possible phases and computing their probabilities. Then transmission probabilities of chromosomal segments from the parents to the progeny must be estimated conditional to the phases. Finally a test statistic (*e.g*. F or likelihood ratio test), based on a given model (*e.g*. regression, mixture model, variance component model...) is performed at each putative QTL position on the chromosomal segments traced. In crosses between inbred lines, the transmission probabilities are simply obtained, as described by [1], from the information given by markers flanking the QTL. In outbred populations, the computation is not straightforward, due to the variability of marker informativity between families and within families between progenies. In [2,3], the transmission probabilities were estimated conditionally to the sole flanking markers. [4-7] used a direct algorithm where all types of gametes corresponding to a linkage group are successively considered: if *L *markers are heterozygous in the parent, 2^*L *^gametes may be produced. This procedure is simple and computationnally fast for a small number of linked markers, but not feasible as soon as their number exceeds about 15. The difficulty can be circumvented in Bayesian approaches using MCMC techniques where these probabilities need not to be explicitly computed (*e.g*. [8]).

Nettelblad and colleagues [9] recently proposed a simple algorithm, which makes the transmission probabilities easily computable even for a large number of markers. In their approach the full length of the linkage group is still considered. A new algorithm, similar to the principle of [9] but exploring the minimum number of useful markers, was implemented in QTLMap software developed by INRA ([10]). Here, we describe and illustrate this algorithm.

### Hypotheses. Notations. Objective

Progeny *p *was born from sire *s *and dam *d*. All were genotyped at *L *loci (*M*_*l*_, *l *= 1 ⋯ *L*). The location of *M*_*l *_on the linkage group, *i.e*. its distance from one end of this group, is *x*(*M*_*l*_) centiMorgan, also denoted *x*_*l*_. The hypothesis of absence of interference is made, allowing the Haldane distance function to be used.

The recombination rate between locus *l*_1 _and *l*_2 _will be noted , *l*_2_. Using the Haldane distance, . When distances vary with sex, the superscript *m *(for males) or *f *(for females) will be used for *x*_*l *_and , *l*_2_.

Let the *l*^*th *^marker information be  for the sire,  for the dam, allele  for the progeny. In *P*_*ilk*_, *i *= *s*, *d *or *p*, the subscript *k *(*k *= 1, or 2) denotes the *k*^*th *^allele read in the records file.

The probabilities of transmission of a chromosomal segment from the parents to the progeny are estimated conditional to parental phases. A phase of parent *i *(*s *or *d*) is characterised by a particular order of its marker phanotypes *P*_*i *_= {*P*_*ilk*_}, for loci *l *= 1 to *L*, giving *G*_*i *_= {*G*_*ilk*_} where *k *= 1 means the grand sire allele and *k *= 2 the grand dam allele. If grand parental origins cannot be built, one of the alleles of the first heterozygous marker in the parent to be phased is arbitrary assigned the subscript *k *= 1.

Let *T*(*M*_*l*_) be the transmission event for marker *l*, and *T*(*M*) the vector of transmission events on the linkage group: *T*(*M*) = {*T*(*M*_1_), *T*(*M*_2_) ⋯ *T*(*M*_*L*_)}. *T*(*M*_*s*_) and *T *(*M*_*d*_) are respectively the transmission events from the sire and from the dam to the progeny. *T*(*M*_*il*_) = *k *if the progeny received *G*_*ilk*_, *i *= *s *or *d*. If the grand parental origins are known, progeny *p *may have received alleles from both its grand sires (*T*(*M*_*sl*_) = 1 and *T*(*M*_*dl*_) = 1, thus *T*(*M*_*l*_) = 11), from its paternal grand sire and maternal grand dam (*T*(*M*_*l*_) = 12), from its paternal grand dam and maternal grand sire (*T*(*M*_*l*_) = 21), or from both its grand dams (*T*(*M*_*l*_) = 22). The probabilities of the transmission events, given the marker phenotypes and parental phases are listed in Table 1 for a biallelic marker.

**Table 1:** *P*[*T*(*M*_*l*_) | *G*_*sl*_, *G*_*dl*_, *P*_*pl*_]: Probabilities of the transmission events, given the marker phenotypes and parental phases, in the case of a biallelic marker (*a, b *alleles)

						*P*(*T*(*M*_***l***_) | *G*_***sl***_, *G*_***dl***_, *P*_***pl***_) for *T*(*M*_***l***_) =			
Case					***P***_***pl***_	11	12	21	22
1	a	b	a	b	(a, a)	1			
2	a	b	a	b	(b, b)				1
3	a	b	b	a	(a, a)		1		
4	a	b	b	a	(b, b)			1	
5	a	b	a	a	(a, a)	1/2	1/2		
6	a	b	a	a	(a, b) or (b, a)			1/2	1/2
7	b	a	a	a	(a, a)			1/2	1/2
8	b	a	a	a	(a, b) or (b, a)	1/2	1/2		
9	a	a	a	b	(a, a)	1/2		1/2	
10	a	a	a	b	(a, b) or (b, a)		1/2		1/2
11	a	a	b	a	(a, a)		1/2		1/2
12	a	a	b	a	(a, b) or (b, a)	1/2		1/2	
13	a	a	a	a	(a, a)	1/4	1/4	1/4	1/4
14	a	a	b	b	(a, b)	1/4	1/4	1/4	1/4
15	a	b	a	b	(a, b) or (b, a)		1/2	1/2	
16	a	b	b	a	(a, b) or (b, a)	1/2			1/2

*G*_*ilk *_is the allele marker *l *the parent *i *is carrying on its *k*^*th *^chromosome ((*k *= (1, 2)); *P*_*pl *_is the marker *l *phenotype of the progeny; *T*(*M*_*l*_) = is the transmission event at marker *l*

The 16 situations described in Table 1 belong to five types:

• Type '*ksd' *: Transmission fully known for both parents (cases 1 to 4),

• Type '*ks*0': Transmission known for the sire only (cases 5 to 8),

• Type '*k*0*d*': Transmission known for the dam only (cases 9 to 12),

• Type '*k*00': Unknown Transmission (cases 13 and 14),

• Type '*amb*': Ambiguous Transmission (case 15 and 16).

The *amb *type corresponds to fully heterozygous trios. It is essential to note that this is the only type of marker phenotypes for which the sire and dam transmissions are not independent (e.g. in situation 15, if sire transmits 1, dam transmits 2 and the reverse).

When the information about one or both parents is missing the conditionnal probability of *T*(*M*_*l*_) most often corresponds to the *k*00 type [1/4, 1/4, 1/4, 1/4]. However, when only one parent possesses a marker phanotype and is phased heterozygous (*a*, *b*), the probabilities are [1/2, 0, 1/2, 0] if *P*_*pl *_= (*a*, *a*) and [0, 1/2, 0, 1/2] if *P*_*pl *_= (*b*, *b*).

Two properties of the transmission probabilities must be underlined:

**Property 1**: Marginally to the marker phenotype, the sire and dam transmission events are independent: *P*[*T*(*M*_*l*_)] = *P*[*T*(*M*_*sl*_)].*P*[*T*(*M*_*dl*_)].

**Property 2**: Due to the no interference hypothesis, the transmission events follow a Markovian process described by:

Note that property 2 is also valid when considering subsets of *M*, *M*_*b *_and *M*_*a*_, allowing an independent estimation of probabilities before and after a given marker *M*_*c*_. If *M *= {*M*_*b*_, *M*_*c*_, *M*_*a*_},

At any position *x *for a QTL, four grand parental origins are possible for the chromosomal segment *Q*_*x *_inherited by the progeny. Let *q *= (*q*_*s*_, *q*_*d*_), (*q *= (11), (12), (21) or (22)), the origin of *Q*_*x*_.

The objective is to estimate *P*_*x*_(*q*) = *P*[*T *(*Q*_*x*_) = *q *| *G*_*s*_, *G*_*d*_, *P*_*p*_], the probability of *q *given the marker information.

To minimize the computation, two procedures are presented: the first one is an iterative exploration of the linkage group, the second a reduction of this group within bounds specific of the tested position *x*.

### Iterative exploration of the linkage group

The observed marker phenotypes and parents' phases can be consistent with different transmission events *T*(*M*). All these events must be considered in turn when evaluating the QTL transmission *T*(*Q*_*x*_). For a given marker transmission event, markers must be successively considered, the no interference hypothesis allowing an iterative estimation of the probability.

**Proposition 1 **: Let Ω be the domain, for the progeny *p*, of transmissions *T*(*M*) consistent with the observations *G*_*s*_, *G*_*d *_and *P*_*p*_. The transmission probability *P*_*x*_(*q*) is given by:(1)

This is obtained after very simple algebra (see appendix).

The domain Ω is obtained listing possible transmissions. If Ω_*l *_is the consistent domain for marker *l*, the Ω domain is formed of nested domains Ω_1 _⊕ Ω_2 _⊕ ⋯ ⊕ Ω_*L*_·Ω_*l *_is directly obtained from Table 1: it is formed of transmission events the probability of which are not nul. For instance, if *G*_*s *_= *aa*, *G*_*d *_= *ab *and *P*_*p *_= *aa*, then Ω_*l *_= {11, 12}.

In the following we shall note *S*_Ω _= ∑_*T*(*M*)∈Ω _*P*[*T*(*M*)] and *T*_Ω _= ∑_*T*(*M*)∈Ω _*P*[*T*(*Q*_*x*_) = *q*, *T*(*M*)].

**Proposition 2 **: The summation *S*_Ω _= ∑_*T*(*M*)∈Ω _*P*[*T*(*M*)] in (1) can be obtained recursively with the following algorithm:(2)

This is obtained under the hypothesis of absence of interference (see appendix).

**Note 1**: the numerator of (1) is obtained similarly, considering the extended domain Ω* = Ω_1 _⊕ Ω_2 _⋯ ⊕Ω_*x *_⋯ ⊕Ω_*L*_, with Ω_*x *_= *q*.

**Note 2**: The *P*[*T*(*M*_*l*_) | *T*(*M*_*l*-1_)] are simply obtained as given in Table 2, for *k *= *l *- 1.

**Table 2:** Transmission probability at locus *l *given the transmission at locus *k*: *P*[*T*(*M*_*l*_) | *T*(*M*_*k*_)]

*T*(*M*_***k***_)	11	12	21	22
*T*(*M*_***l***_)				
11				
12				
21				
22				

is the recombination rate for sex *i*, between loci *l *and *k*.

They may be summarized by a single formulae. Let *θ*⟨*r*, *i*, *j*⟩ = 1 - *r *- (1 - 2*r*).(*i *- *j*)^2^,

**Note 3**: System (2) may be generalized to any subdivision of the linkage group *M*, *M *= {*M*_1_, *M*_2_, ⋯ *M*_*G*_}, defining *T*(*M*_*g*_), *g *= 1 ⋯ *G*, as the vector of *T*(*M*_*l*_), *l *∈ *M*_*g*_.

### Reduction of the linkage group

The set of markers *M *= {*M*_*l*_, *l *= 1 ⋯ *L*} may be sequenced as *M *= {*M*_*a*_, *M*_*α*_, *M*_*c*_, *M*_*β*_, *M*_*b*_} where *M*_*c *_is a subset of interest, *M*_*β *_and *M*_*α *_its flanking markers, and *M*_*b *_and *M*_*a *_all the remaining markers before and after the area (*M*_*α*_, *M*_*c*_, *M*_*β*_). We now propose three simplifications of the summation *S*_Ω _= ∑_*T*(*M*)∈Ω_*P*[*T*(*M*)].

**Proposition 3 **: In the summation *S*_Ω_, the type *k*00 markers can be ignored, *i.e*. they may be bypassed in the iterative system (2).

Here *M*_*c *_is a single *k*00 type marker. Proposition 3 means (see appendix for a demonstration) that, in (2), the sequence:

which corresponds to two iterations, may be replaced by:

**Proposition 4**: In the summation *S*_Ω_, the elements corresponding to the unknown parental transmission for types *k*0*d *or *ks*0 markers can be ignored, *i.e*. they may be bypassed in the iterative system (2).

Here *M*_*c *_is a single *ks*0 or *k*0*d *type marker. Proposition 4 means (see appendix for a demonstration) that, in (2), the sequence

which corresponds to two iterations, may be replaced by (successively *k*0*d *and *ks*0 markers):

**Corollary 1**: In the summation *S*_Ω_, a sequence *M*_*c *_of markers all belonging to "*k*" types (*i.e*. non *amb*) appears as a single element where only the certain transmissions are involved.

From propositions 3 and 4,

where the markers subscripted *j*_*s *_(= 1 ⋯ *J*_*s*_) are successive markers belonging to *ksd *or *ks*0 types, and the markers subscripted *j*_*d *_(= 1 ⋯ *J*_*d*_) to *ksd *or *k*0*d *types in the sequence *M*_*c*_.

**Definition **: A series of markers *N *= {*M*_*α*_, *M*_*c*_, *M*_*β*_} starting with a *ks*0 (*resp. k*0*d*) type marker {*M*_*α*_}, ending with a *k*0*d *(*resp. ks*0) type marker {*M*_*β*_}, and only with *k*00 type markers between those bounds (in *M*_*c*_) will be called a sd-node (*resp*. ds-node).

**Proposition 5**: If the sequence *N *= {*M*_*α*_, *M*_*c*_, *M*_*β*_} of *M *is a sd-node, the summation *S*_Ω _may be separated in three terms corresponding to [*M*_*b*_/*M*_*βs*_, *M*_*αd*_], [*M*_*βs*_, *M*_*αd*_], and [*M*_*a*_/*M*_*βs*_, *M*_*αd*_] Proposition 5 means (see appendix for a demonstration) that, in (2), *S*_Ω _is obtained by

**Note 4**: The {*M*_*β*_, *M*_*c*_, *M*_*α*_} sequence may be reduced to a single marker *M*_*γ *_if it belongs to the *ksd *type. In this case,

In general we shall note *T*(*N*) the transmission event for a node, {*T*(*M*_*sβ*_), *T *(*M*_*dα*_)}, {*T*(*M*_*dβ*_), *T*(*M*_*sα*_)} or *T*(*M*_*γ*_).

**Corollary 2**: If the tested QTL position *x *is located in segment *M*_*c *_between two nodes *N*_1 _and *N*_2_, only the markers belonging to the interval [*N*_1_, *N*_2_] have to be considered when computing the transmission probability *P*[*T*(*Q*_*x*_) = *q *| *G*_*s*_, *G*_*d*_, *P*_*p*_], see appendix, giving:(3)

### Algorithm

Based on the propositions and corollaries developed above, an algorithm for the computation of transmission probabilities of the chromosomic segment *x *can be given.

1. From the position *x*, the markers are explored towards the left until a node (a *ksd *type marker or a pair of markers one of *ks*0 and the other of *k*0*d *type, separated only by *k*00 type markers) or the extremity of the linkage group is found. Let *T*(*N*_*l*_) be the transmission events for the left node *N*_*l*_. *P*[*T*(*N*_*l*_)] = 1/4.

2. From the position *x*, the markers are explored towards the right until a node or the extremity of the linkage group is found. Let *T*(*N*_*r*_) be the transmission events for the right node *N*_*r*_. *P *[*T *(*N*_*r*_)] = 1/4. The only necessary informative segment for *x *in the full linkage group is {*N*_*l*_, *N*_*r*_}.

3. Let  the *amb *type markers in {*N*_*l*_, *N*_*r*_}. Together with *N*_*l *_and *N*_*r*_, the  delimit *n *+ 1 intervals *I*_*k*_, which may be empty or include *k*00, *ks*0 or *k*0*d *type markers. The reduced summation , see (the part of *S*_Ω _which differs from *T*_Ω _and has to be used in  see appendix) is computed iteratively:(4)

It must be underlined that there is no node between two adjacent *amb *type markers of the informative segment {*N*_*l*_, *N*_*r*_}, since this segment ends at the first node found on both sides. As a consequence, neither a *ksd *marker type nor a mixture of *ks*0 and *k*0*d *types markers could be found between the ambiguous markers *M*(*a*_*k*_) and *M*(*a*_*k*+1_): the *I*_*k *_interval may be classified as *K*00 (only *k*00 types markers), *Ks*0 (one or more *ks*0 type markers, no *k*0*d *type marker and any number of *k*00 type markers) or *K*0*d *(the reverse).

4. Let  and  be two successive *amb *markers, in the iterative process (4), the probabilities *P *[*T*()/*T*()] are given by

where *θ*⟨*r*, *i*, *j*⟩ = 1 - *r *- (1 - 2*r*).(*i *- *j*)^2^.

5. The reduced summation  is computed iteratively adding the *T*(*Q*_*x*_) transmission in the list of transmission {*T*[*N*_*l*_], *T*[], ⋯, *T*[], *T*[*N*_*r*_]}.

6. The transmission probability *P*[*T*(*Q*_*x*_) = *q *| *G*_*s*_, *G*_*d*_, *P*_*p*_] = .

**Note 5 **: The algorithm can be organised scanning the interval {*N*_*l*_, *N*_*r*_} from the left to the right rather than from the right to the left as described above.

### Example

**Figure 1:**
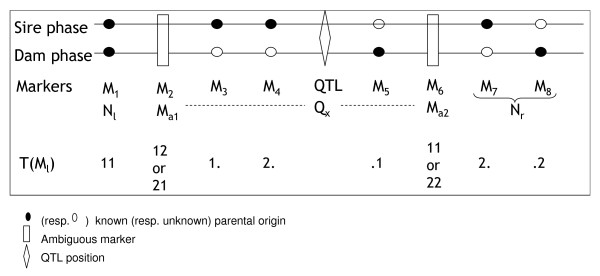
**Example of a linkage group with 8 markers including 2 ambigous**. The figure represents a chromosome with eight markers. Two (*M*_2 _and *M*_6_) are ambiguous (For *M*_2_, the progeny received either the 1^*st *^allele of its sire and 2^*nd *^allele of its dam, or the 2^*nd *^of its sire and 1^*st *^of its dam. The nodes are, on the left, the first marker, and on the right, markers *M*_7 _and *M*_8_. The dark (respectively white) circles represent markers with a known (respectively unknown) grand parental origin.

A linkage group of eight markers is available (Figure 1). Markers *M*_2 _and *M*_6 _are ambiguous, with types 15 and 16. Markers 1 and 8 are fully informative (types 1 and 2), the other markers are semi informative. The tested position for the QTL *x *is located between markers 4 and 5. The nodes are, on the left, marker 1 (*ksd *type) and on the right, the group *M*_7 _- *M*_8_. Thus the informative segment here is the full group. Steps of the proposed algorithm are detailed Table 3.

**Table 3:** Calculation of the marker transmission probability corresponding to the example in Figure 1

*T*(*N*_***l***_)	11			
*P*[*T*(*N*_***l***_)]	1/4			
*T*()	12	21	12	21
*P*[*T*()|*T*(*N*_*l*_)]				
*F*[*T*()|*T*(*N*_*l*_)]				
*T*()	11	11	22	22
*P*[*T*()|*T*()]				

*F*[*T*()]				
*P*[*T*(*N*_*r*_)|*T*()]				

*F*[*T*(*N*_*r*_)]				

## Discussion - Conclusion

The algorithm presented in this paper to estimate the transmission probability of QTL from parents to progeny needs only very limited computational resources, both in terms of time and space. Complementary to the algorithm presented by Nettleblad and colleagues (2009), it limits the exploration of the linkage group to the markers really informative for a given position to be traced, and thus performs faster. As [9], it deals with sex differences between recombination rates.

The QTL transmission probability is estimated conditionnaly to the observed transmission at the surrounding markers loci. The algorithm does not make use of possible information about the marker allele frequencies to fill potential information gaps.

The major difficulty addressed in this algorithm is the non independence of transmission events from the sire and the dam to the progeny in triple heterozygous trios. In the absence of such trios, the transmission from the parents are fully independent and may be treated separately simply by considering the flanking informative markers. This is the case for QTL located on the sex chromosome X or W.

The algorithm has been developed in the framework of QTL detection designs involving two or three generations in outbred populations. It has been implemented in QTLMap, a software for the analysis of such designs. QTLMap is available upon request to the authors.

In more complex pedigrees, the transmission probability should not be conditioned only on parents phases and progeny marker phanotypes. Information from the grand progeny (and the spouses lineages) may improve the estimation, since the progeny phase can be inferred, at least partially, from these data. A recursive process inspirated from [3] should possibly be implemented.

The transmission probabilities are estimated conditionally to parental phases. In linear approaches (*e.g*. the Haley Knott regression), if more than one phase is probable, the marginal transmission probability could be estimated considering all of them in a weighted sum of conditional probabilities. Alternatively, the only most probable phase could be considered [11].

The absence of interference hypothesis is central in the present algebra. If this is not true, then most of the propositions are not valid and the algorithm not applicable.

Finally, compared to the most common codominant markers, dominant markers will be characterized by a lower informativity, with an increase of the between nodes segment length and a concomitant decrease of the transmission probability.

## Competing interests

The authors declare that they have no competing interests.

## Authors' contributions

JME drafted the manuscript. All authors participated in the development of the method and read and approved the final manuscript.

## Appendix: Demonstration of the propositions and corollary

**Proposition 1: ***P*[*T*(*Q*_*x*_) = *q *| *G*_*s*_, *G*_*d*_, *P*_*p*_] = 

And, similarly, *P*[*T*(*Q*_*x*_) = *q*, *P*_*p *_| *G*_*s*_, *G*_*d*_] = *P*[*T*(*Q*_*x*_) = *q*, *T*(*M*)] if *T*(*M*) ∈ Ω, = 0 if not

Proposition 2

Due to the no interference hypothesis, the transmission events follow a Markovian process described by:

Thus

The summations may be inverted:

Consequently:

Proposition 3

With an argument similar to the demonstration of proposition 2, the sum *S*_Ω _may be expressed as:

Thus(A1)

As Ω_*c *_forms a complete set of events, since all transmissions are possible,

Thus

Proposition 4

In the equation(A1), we have, from property 1,

Without loss of generality, we assume that the parent with unknown transmission at *M*_*c *_is the sire. There is a unique consistent *T*(*M*_*dc*_), and the 2 possible *T*(*M*_*sc*_) form a complete set of events, thus:

The simplification of *F*[*T*(*M*_*β*_)] follows:

Proposition 5

When *M*_*c *_contains markers of *k*00 type, they can be forgotten following proposition 3. We thus assume that the *M*_*c *_group is empty, and the linkage group is described as *M *= {*M*_*b*_, *M*_*β*_, *M*_*α*_, *M*_*a*_}

But *P*[*T*(*M*_*b*_), *T*(*M*_*dβ*_) | *T*(*M*_*sβ*_), *T*(*M*_*α*_), *T*(*M*_*a*_)] = *P*[*T*(*M*_*b*_), *T*(*M*_*dβ*_) | *T*(*M*_*sβ*_), *T *(*M*_*dα*_)]

Thus

Corollary 2

Let *M *= {*M*_*b*_, *N*_*l*_, *M*_*c*_, *N*_*r*_, *M*_*a*_*}*, with *x*(*N*_*l*_) ≤ *x *≤ *x*(*N*_*r*_)

From proposition 5, assuming both nodes *N*_*l *_and *N*_*r *_are sd-nodes,

From proposition 5 again,

The elements  and  being also present in the numerator *T*_Ω _of (1) they can be forgotten.

The summation *S*_Ω _may be reduced to :

Similarly
